# Dual wavelength spread-spectrum time-resolved diffuse optical instrument for the measurement of human brain functional responses

**DOI:** 10.1364/BOE.393586

**Published:** 2020-06-02

**Authors:** Konstantinos I. Papadimitriou, Ernesto E. Vidal Rosas, Edward Zhang, Robert J. Cooper, Jeremy C. Hebden, Simon R. Arridge, Samuel Powell

**Affiliations:** 1 Department of Computer Science, University College London, London, WC1E 6BT, UK; 2 Department of Medical Physics and Biomedical Engineering, University College London, London, WC1E 6BT, UK; 3 Faculty of Engineering, University of Nottingham, Nottingham, NG7 2RD, UK; 4 These authors contributed equally to this work

## Abstract

Near-infrared spectroscopy has proven to be a valuable method to monitor tissue oxygenation and haemodynamics non-invasively and in real-time. Quantification of such parameters requires measurements of the time-of-flight of light through tissue, typically achieved using picosecond pulsed lasers, with their associated cost, complexity, and size. In this work, we present an alternative approach that employs spread-spectrum excitation to enable the development of a small, low-cost, dual-wavelength system using vertical-cavity surface-emitting lasers. Since the optimal wavelengths and drive parameters for optical spectroscopy are not served by commercially available modules as used in our previous single-wavelength demonstration platform, we detail the design of a custom instrument and demonstrate its performance in resolving haemodynamic changes in human subjects during apnoea and cognitive task experiments.

## 1. Introduction

Near-infrared spectroscopy (NIRS) is an optical technique that uses non-ionising electromagnetic radiation to extract meaningful information in a wide range of industrial and biomedical applications. In the field of medical imaging, NIRS has seen a remarkable upsurge of interest in the last two decades. Near-infrared light (in the range of 0.6-1 µm for medical applications) has the ability to penetrate up to several centimetres in biological tissues, since in this wavelength range absorption is relatively low, and scattering interactions dominate [1]. NIRS has predominantly been used to provide clinicians with qualitative and quantitative, real-time information about patients’ oxygenation levels, by measuring the levels of the chromophore haemoglobin [2]. The ability to evaluate concentration changes in oxygenated haemoglobin (${\mathrm{HbO}}_{2}$), de-oxygenated haemoglobin (HHb), total haemoglobin (tHb) and oxygen saturation (${\mathrm{SO}}_{2}$) in tissues, combined with the non-invasive nature of this method, which allows for bedside monitoring, has established NIRS as a powerful clinical tool for brain, muscle or breast monitoring [3].

The association between functional activation and cerebral oxygenation levels permits *functional* NIRS (fNIRS), which has attracted significant attention [2,4–6] over the past two decades. As a result of this interest a large number of instruments and techniques has been proposed, each relying upon a different light emission and detection method, with their own advantages and disadvantages [1]. However, all NIRS devices, fall into three main interrogation technique categories: (i) continuous wave (CW); (ii) frequency domain (FD); (iii) time domain (TD) [7–9]. Through the CW modality, the light attenuation of a constantly illuminated tissue is monitored, while in FD the sample is illuminated with intensity-modulated light and both the attenuation and phase delay of emerging light is recorded. In the TD modality, an ultra-short light pulse (circa few picoseconds pulse width) is injected into the turbid medium and the photon distribution of the time-of-flight (DToF) is measured at the detector using single photon counting techniques [4,10,11]. The facility of TD NIRS to measure photon pathlengths in biological tissue enables the accurate recovery of absolute optical properties, and greater depth sensitivity and specificity when used in diffuse optical tomography (DOT) applications [12,13].

Despite the fact that CW instruments are capable of measuring only the intensity of the diffuse light, and consequently light scattering and absorption effects can not be easily differentiated, CW NIRS is the most commercially exploited technique to date, primarily due to the instruments’ simplicity, small size and low cost. In contrast, TD NIRS instruments are bulky and expensive and it appears that these factors have limited the expansion of this technique beyond specialist clinical or research environments, despite its superiority compared to the other two methods. Thus, an instrument that combines the advantages of the TD technique with a low-cost, small-footprint setup could allow for richer and more robust approaches to human brain mapping.

In our previous research, we have demonstrated the potential of an alternative technique for TD measurements, relying upon the spread spectrum paradigm for ToF-resolved measurements [14,15]. The spread spectrum approach to (f)NIRS operates by the same principle as has been employed in telecommunications and radar over the past 50 years [16]. The input pulse is broadened in time by modifying the phase of its Fourier components, such that the signal to be transmitted has a considerably lower peak-power than the original pulse, but with the same frequency content. Following transmission through a linear time-invariant system, the same transformation is applied in reverse to reconstruct the response which would have been received had a pulse been applied. Whilst there are many methods by which to temporally broaden the input pulse, we seek to employ a binary signal such that an all-digital approach can be employed, avoiding the cost and complexity associated with high bandwidth analogue electronics. Accordingly, we use a maximum-length sequence (MLS) as our temporally broadened pulse, owing to its excellent autocorrelation properties. This sequence is transmitted optically to the subjects under test, and the diffused light is collected based on standard time correlated single photon counting (TCSPC)-based detection techniques. Following cross-correlation of the detected signal with the transmitted input sequence, the system’s impulse response function (IRF) or temporal point spread function (TPSF) [14,15] are recovered. A related approach has also been taken by other groups [17,18] where the demodulation at a particular point in time, or in the domain of the Laplace transform, is performed in the analogue electronics prior to sampling; this has the advantage of reducing the requirements for data acquisition and processing. The key advantage of the spread-spectrum approach is that it allows the use of low-power sources for tissue illumination, such as vertical-cavity surface-emitting lasers (VCSELs), which are significantly cheaper and more compact than traditional pulsed excitation lasers. Whilst the spread-spectrum method fundamentally leads to a higher noise floor and reduced dynamic range compared to traditional pulsed excitation systems, we have previously shown that the recorded time-of-flight information is of sufficient quality to enable its use in forming quantitative measures of haemodynamic changes in typical experimental paradigms. The stability and pertinent aspects of the temporal performance, such as jitter, was shown to be comparable with traditional pulsed systems demonstrated in the literature [4,19–21].

In our previous work we demonstrated a single wavelength system which used a commercially available 850nm optical transceiver designed for telecommunications. A single wavelength does not permit computation of the chromophore concentrations of clinical significance, and alternative wavelengths are not routinely commercially available. In this work we demonstrate the design and implementation of a new system based upon the spread-spectrum technique, developing our own optical transceivers and optical components to enable dual-wavelength operation, optimised for use in NIRS applications. This enables us to demonstrate the measurement of functional responses during cognitive experiments. The paper is structured as follows: in Section 2, the implementation of our dual-wavelength instrument for fNIRS applications is described. In Section 3, *in vivo* experimental results are presented from three healthy subjects, participating in two types of experiments: (a) an apnoea and (b) a neurocognitive task. All experiments are accompanied by quantitative haemodynamic responses, and whilst not the focus of this work we have also examined the form of time-domain measures such as the mean ToF, intensity and phase, and time-gated measurements, since these may be used in the future for enhanced sensitivity at depth. Finally, a detailed discussion of the potentials and future improvements of the proposed setup is offered.

## 2. Spread spectrum fNIRS system description

### 2.1 System configuration

**Fig. 1.**
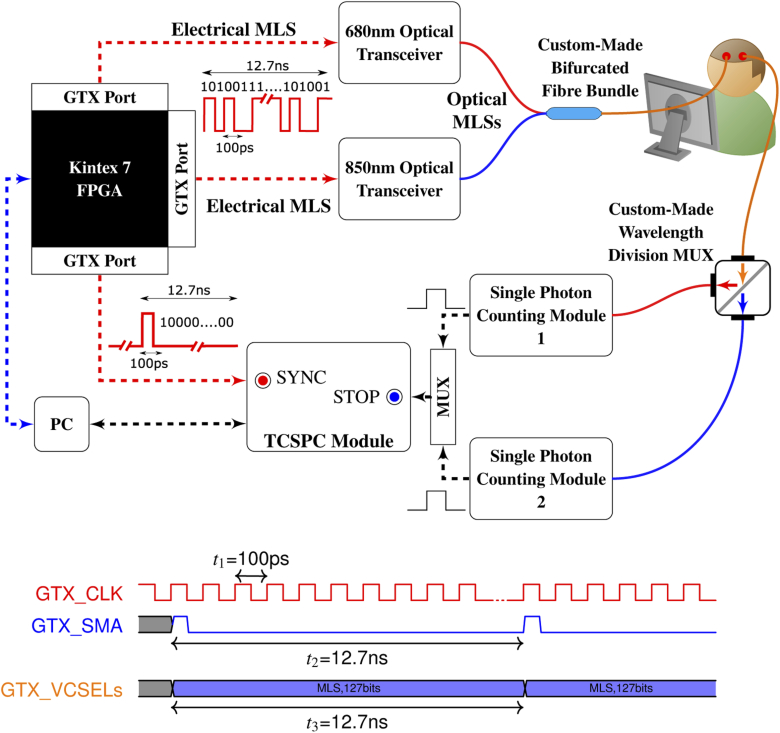
A schematic diagram of the dual-wavelength spread spectrum-based TD system for fNIRS applications. The timing diagram at the bottom of the Figure indicates the synchronous transmission of all FPGA’s GTX transmitter ports.

A schematic representation of our new dual-wavelength spread spectrum-based TD instrument is shown in Fig. 1. Two custom-made optical transceivers have been designed and fabricated, hosting a 680 nm (680C-0000-B002, VIXAR Inc.) and a 850 nm (I0-0850M-0000-B002, VIXAR Inc.) communications-grade multi-mode VCSEL, respectively. Each VCSEL is supplied in a TO-can package for free-space operation. Fibre coupling was achieved by removing the can and locating a lensed 50/125 µm (core/cladding) multi-mode fibre in front of the VCSEL. The assembly is then hermetically sealed, the fibre protected with with 1mm PVC tubing, and a compact ∼8×16 mm heatsink installed to promote heat dissipation.

The optical transceivers have been fabricated on a 4-layer, 1 mm thickness printed circuit board (PCB) with dimensions 12×63 mm. One end of both PCB has been designed to mate directly with the industry standard 20-contact SFP/SFP^+^ electrical connector, incorporating the required power, ground and signal connections. The radio-frequency (RF)-performance PCBs have been further optimised using impedance control techniques, taking into consideration the required differential input resistance of the VCSEL driver and the resistance of the VCSELs. A low-power integrated limiting amplifier and VCSEL driver integrated circuit (IC)(MAX3798, Maxim) has been used to drive both VCSELs at 10 Gb/s by providing appropriate modulation and bias currents, both fully controllable via a 3-wire digital interface. The VCSEL driver IC can provide modulation current up to 12 mA and bias current up to 15 mA, with deterministic jitter <6 ps and rise and fall times at the transmitter output down to 26 ps [15]. A summary of the properties of the VCSEL sources of the instrument can be found in Table 1.

**Table 1.** Properties of the VCSEL light sources of the instrument.

λ(nm)	Δλ(nm)	Slope Effcy(mW/mA)	${\text{P}}_{\text{Out}}$(mW)	${\text{I}}_{\text{Mod}}$(mA)	Fibre Core (μm)
680	2.1	0.8	2.7 (max)	15^*a*^	50
850	10	0.85	10 (max)	20^*a*^	50

^*a*^ Pulsed current

Each VCSEL is modulated at 10 Gb/s by the Multi-Gigabit Transceivers of a Kintex 7 FPGA (KC705, Xilinx), called GTX Transceivers. Three of the sixteen GTX ports of Kintex 7 FPGA have been employed, all programmed to operate at 10 Gb/s in a synchronous manner, clocked by the same GTX reference clock. The two GTX ports modulating the two optical transceivers transmit the same fixed length MLS electrical signal which is converted to an optical signal by the transceivers. An MLS with length NMLS=2^q^ −1 and q=7 has been chosen corresponding to a binary sequence with 12.7 ns period. This period is sufficiently long to allow recovery of the TPSFs and IRFs, which are typically a few ns in length. The third GTX port transmits a single 100 ps period electrical pulse with the same period as the MLS. This signal is used as the **SYNC** signal of the TCSPC card (SPC-130, Becker & Hickl). A quad SFP/SFP^+^ transceiver FMC (VITA 57) module (Faster Technology, LLC.) has been used to host both optical transceivers to the FPGA board. The current FPGA board, together with the FMC expansion module, can host up to five optical transceivers (sources).

The two fibre-coupled VCSELs are connected to a custom-made 50/125 µm (core/cladding), 2×1, fibre bundle fabricated at University College London. The bundle is responsible for combining the two wavelengths, which are later injected into the subjects under test. The combined diffused light is collected from the subject under test using a 50/125 µm fibre, which is connected to the input port of a custom wavelength division multiplexer (WDM) (WDM-12P-111-680/850-50/125, OZoptics Ltd). The WDM collimates the diffuse light which is then split by a dichroic filter, with the longer wavelength transmitted from output port T, while the shorter wavelength is reflected from output port R. Finally, the transmitted and reflected beams of the two output ports are focused into two 50/125 µm (core/cladding) fibres, which are subsequently directly coupled to the two single photon counting modules (SPCMs) of the setup. Both SPCMs (SPCM-AQRH-14-FC, PerkinElmer) feature a thermoelectrically cooled and temperature controlled silicon photon avalanche diode (SPAD), specified as having an active area of 175um, timing resolution of 350ps, and a quantum efficiency of 45% at 850nm. We note that the literature suggests that the timing resolution of these SPCMs increases significantly at higher count rates, with a full-width half maximum in the order of ∼600ps at the count rates experienced in our work [22]. The TTL signals from the SPCMs’ outputs act as the **EVENT** electrical signals for the TCSPC acquisition card. Because the SPC-130 is a single channel TCSPC card, an 8-channel router (HRT-81, Becker & Hickl) is used to multiplex to both high-speed **EVENT** signals [14]. Finally, the collected raw TCSPC data are post-processed according to our previous work [14,15].

### 2.2 System performance

**Fig. 2.**
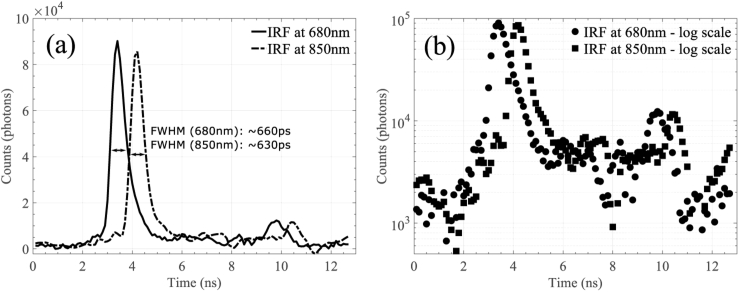
The resulting IRFs of the instrument for both wavelengths after cross-correlation with the transmitted MLSs in linear-(left) and log-scale (right).

Indicative system IRFs for both wavelengths in linear- and log-scale are shown in Fig. 2. The FWHM has been computed for each wavelength and it has been found to be ∼660 ps and ∼630 ps for the 680 nm and 850 nm source, respectively. The presented IRFs indicate that the performance of the custom-made optical transceivers is comparable to the performance of commercially available optical transceivers, with respect to speed and noise properties [14,15]. The log-scale representation of both IRFs, shown in Fig. 2(b), reveals that for both wavelengths a distinct second peak appears, at around ∼10 ns after the primary peak. This peak is likely to be due to a reflection occurring during the IRF measurement. As discussed in the introduction, the dynamic range of our system is roughly one or two orders of magnitude lower than other reported traditional TD NIRS instruments [4,9], with a noisier baseline.

The stability and linearity of the spectroscopic system has been thoroughly investigated and shown in our previous work [15]. The system is capable of achieving intensity variations of ±0.5%, while the relative mean ToF stability is less than ±15 ps. Intensity and mean ToF stability can be maintained for long time periods (up to 8 hours have been continuously monitored) without exhibiting any instabilities. As already pointed in [14,15], the instrument operates at these ranges, following an initial *warm-up* period of ∼40 minutes, a period significantly smaller compared to traditional pulsed-laser-based TD setups. Linearity and accuracy of the VCSELs have been investigated in our previous work [15] and the results were comparable to traditional TD systems.

## 3. *In vivo* experimental results

Our upgraded system’s performance was evaluated by employing two different types of *in vivo* experiments. The first was an apnoea (breath holding) test, which, in principle, triggers a global haemodynamic response to the subjects [23]. The second was a neurocognitive task, called the symbol digit modalities test (SDMT), which triggers haemodynamic changes to the prefrontal cortex of the subjects [24,25]. Three healthy, adult male subjects participated in both experiments and the group’s average haemodynamic and TD-related responses were calculated. In both experiments, the source and detector fibres were placed and centred over the left prefrontal cortex area (F3 of the 10/20 EEG system), with a 30 mm separation. The fibres were stabilised on the subjects’ forehead using flexible, custom-made, 3D-printed base (TangoBlack^+^ material printed by a Stratasys Objet Connex 500 3D printer) with acrylic ferrules for further fibre stabilisation. In addition, bandages were placed around the subjects’ head and the 3D-printed base, to ensure a fixed position between the source and detector fibres. The experiments were performed in dark room conditions and all subjects were in a comfortable, seated position. The integration time for each TPSF measurement was set equal to ∼0.3 seconds to avoid aliasing of heartbeat. Finally, all haemodynamic and TD signals were filtered using a moving average algorithm (LOESS), implemented in Matlab (Mathworks, Inc.), which allowed us to remove any unwanted physiological rhythms that could interfere with the signals. For all the experimental results shown below, 5 TPSFs were averaged for each data point, resulting in a total integration time of ∼1.5 seconds. In both experiments the HHb and ${\mathrm{HbO}}_{2}$ concentration changes were computed for each subject and across group averages.

### 3.1 Data preprocessing

The concentration values of HHb and ${\mathrm{HbO}}_{2}$ were computed using the modified Beer-Lambert law, based on the measured intensity and mean ToF changes for both wavelengths during the experiment. The differential pathlength factor (DPF) has been determined for each specific subject separately, via measurement of the mean time taken for light to traverse the distance from source to detector on the head, as explained in detail in the literature [7,8,26]. The total time of each experiment, defined as T = $\left\{t_{1},t_{2},\ldots t_{F}\right\}$, can be further distinguished into three separate phases, i.e. $t^{\text{base}}$, $t^{\text{act}}$ and $t^{\text{rec}}$, denoting the baseline, activation and recovery phases of the tasks. A measure of intensity with our system can be computed via two different methods, as also shown explicitly in literature [4,7,27]. The first method resembles a CW-based intensity calculation, which relies upon the sum of photons collected in a specific time window of the experiment. More specifically, for experimental time $t_{j}$, the count of all photons in the window is defined as: ${\text{N}}_{j}=\sum$(photons), arriving in $t\;\in \;[t_{j},t_{j+1}]$. The second method for intensity calculation relies upon the recovery of the various TPSFs throughout the experiment and the Mellin-Laplace transform, whose general form is shown in the equation below:

(1) $$M_{j}^{p,u}[\Gamma_{j}(t)]=\frac{\int_{t_{k}=T_{0}}^{t_{k}=T_{f}}\Gamma_{j}(t)t^{u}e^{-pt}\text{d}t}{\int_{t_{k}=T_{0}}^{t_{k}=T_{f}}\Gamma_{j}(t)\text{d}t},$$

where the values of the variables u and p determine the temporal moments and/or the rate of decay of Laplace coefficient of the transform and subsequently correspond to different data types [7,8,18,28]. $\Gamma_{j}(t_{k})$ denotes the TPSF at each specific time window, defined (theoretically) as:

$$\Gamma_{j}(t_{k})=\text{sum of photons arriving in bin}\;(\text{width}\;\Delta t)\;\;t\in t_{j,k}\in [t_{j+k\Delta t},t_{j+(k+1)\Delta t}].$$

It is worth recalling that with our spread spectrum-based instrument we do not directly record the TPSF as in a conventional TCSPC technique. Instead, we infer Γ(t) from cross-correlation with the ideal, binary transmitted MLS [14,15]. However, this intermediate post-processing step allows us to extract the traditional TPSF and then proceed with the necessary photon count of each time bin. Based on the extensive analysis shown in [7,8,28], by setting both variables u and p of the Mellin-Laplace transform to 0, the *integrated intensity* or $0^{\text{th}}$-moment can be calculated. Similarly, by setting variables u and p of the transform to 1 and 0, respectively, the *mean ToF* or $1^{\text{st}}$-moment of the produced TPSF for each time bin can be computed. Below, a summary of the equations used to calculate the haemodynamic and TD responses is provided.

(2) $$\Delta {\text{Intensity}}_{j}^{\text{CW},\lambda }=-\ln\left[N_{j}^{\text{T},\lambda }/N_{j}^{\text{base},\lambda }\right]$$

(3) $$0^{\text{th}}{\text{moment}}^{\text{TPSF},\lambda }=-\ln\left[\frac{\int_{t_{k}=T_{0}}^{T_{f}}\Gamma_{j}^{\text{T},\lambda }(t_{k})\text{d}t_{k}}{\int_{t_{k}=T_{0}}^{T_{f}}\Gamma_{j}^{\text{base},\lambda }(t_{k})\text{d}t_{k}}\right]$$

(4) $$1^{\text{st}}{\text{moment}}^{\text{TPSF},\lambda }=\frac{\int_{t_{k}=T_{0}}^{T_{f}}\Gamma_{j}^{\text{T},\lambda }(t_{k})t_{k}\text{d}t_{k}}{\int_{t_{k}=T_{0}}^{T_{f}}\Gamma_{j}^{\text{base},\lambda }(t_{k})\text{d}t_{k}}$$

Whilst Eq. (2) describes intensity as the (negative log of) sum of photons at each individual time point, normalised over the mean of the baseline intensity, Eq. (3) records the intensity measure from Γ(t), as recovered from the reconstructed TPSF procedure. The two measures should (and will be shown to) produce the same results, though the noise statistics may differ. The negative logarithm added to Eq. (2) and Eq. (3) complies with the definition already shown in [4,27]. The reader should also note that the mean ToF or $1^{\text{st}}$-moment shown in Eq. (4) is normalised by integrated intensity, thus, it is independent of variations in intensity.

Where we consider intensity and phase at particular frequencies, these are acquired by discrete Fourier transform of the recovered TPSF. Finally, for the cognitive SDMT experiment, contrast was computed for two different time-gates of the TPSF, using Eq. (3), for both wavelengths.

### 3.2 Apnoea experiment

All subjects followed the same experimental protocol, which comprises: (i) a 7 seconds baseline; (ii) a 30 seconds breath-holding period; and (iii) a 50 seconds recovery time. For each subject, 10 repetitions were performed and averaged. Before the start of each breath-holding period of the protocol, the subjects were asked to exhale completely, in order to remove as much air as possible from their lungs, in an attempt to emulate as faithfully as possible the apnoea responses. The subjects were instructed when to hold their breath and when to start breathing again. Each phase of the experiment was timed by a second person in the room, in order to generate an identical time protocol for all subjects, in all their repetitions.

**Fig. 3.**
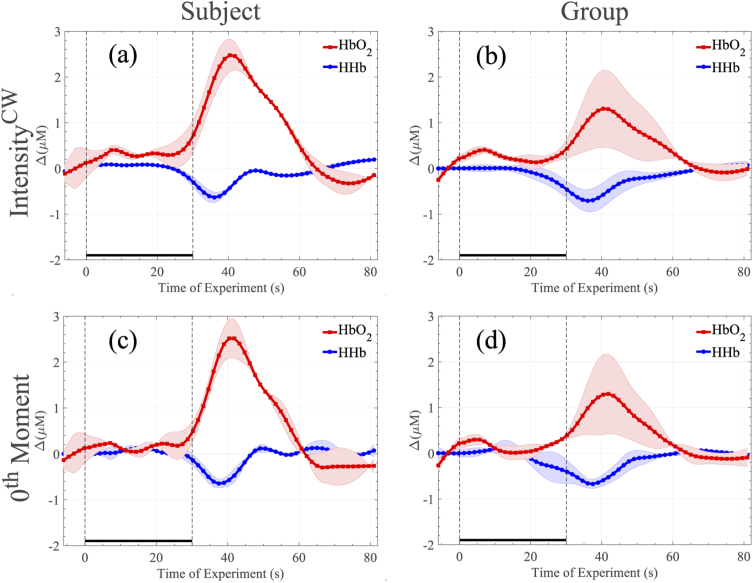
Haemodynamic responses of the apnoea experiment. In (a) and (c) the haemodynamic responses of an individual subject over 10 repetitions is shown while in (b) and (d) the group’s average is demonstrated. Figures (a) and (b) have been produced by calculating intensity as the total number of photons collected for each wavelength at each sample point. Figures (c) and (d) are based on the $0^{\text{th}}$-moment definition. All changes have been plotted with respect to the baseline, defined as the beginning of the experiment. The two dashed vertical lines represent the beginning and end of the activation period.

The haeomodynamic responses generated in this experiment are summarised in Fig. 3. Figures 3(a) and (c) demonstrate the changes of an individual subject, computed based on Eq. (2) and Eq. (3), respectively. Figures 3(b) and (d) present the average changes of HHb and ${\mathrm{HbO}}_{2}$ concentrations of the group, computed based on Eq. (2) and Eq. (3), respectively. Both methods, in both cases, generated almost identical haemodynamic responses, as expected.

During the 30 s breath-holding period, ${\mathrm{HbO}}_{2}$ and HHb remained almost constant, when the subjects start breathing again, we can start to see the variation (increase in ${\mathrm{HbO}}_{2}$ decrease in HHb), due to increased oxygen supply. This response is consistent with that expected due to the action of cerebral autoregulation, and differs from the systemic response expected in a bulk change during, e.g., cuff occlusion [29].

**Fig. 4.**
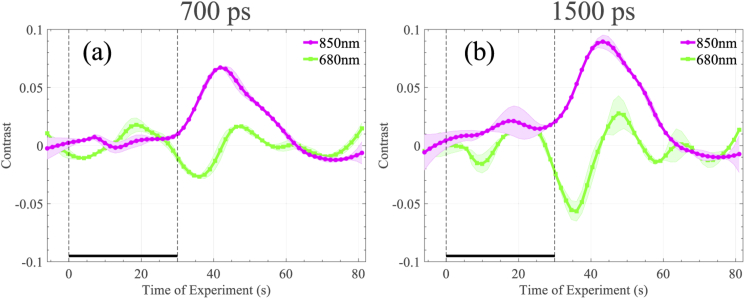
Contrast at 680 and 850nm for different time-gates, utilising a fixed width window equal to 300 ps. (a) demonstrates the contrast of an indicative subject during the apnoea experiment with 700 ps delay, while (b) shows contrast response of the same subject in the same experiment when the delay is 1500 ps. Average and standard deviation over 10 repetitions is shown in both cases.

We also chose to inspect the intensity contrast at different time-bins of the TPSF during the experiment. To generate these results a 300ps window was applied to all of the TPSFs collected during one instance of the experiment, for all ten repetitions. In Fig. 4(a) we present the contrast results for an early time gate of ${\text{t}}_{d}$ = 700 ps, and in Fig. 4(b) we present the same measurement for a late time gate of ${\text{t}}_{d}$ = 1500 ps case. Contrast has been computed based on the integrated intensity shown in Eq. (3). Both time gates demonstrate a similar profile, though the contrast in the later gate is greater, suggesting sensitivity to deeper regions probed by the near infrared light.

**Fig. 5.**
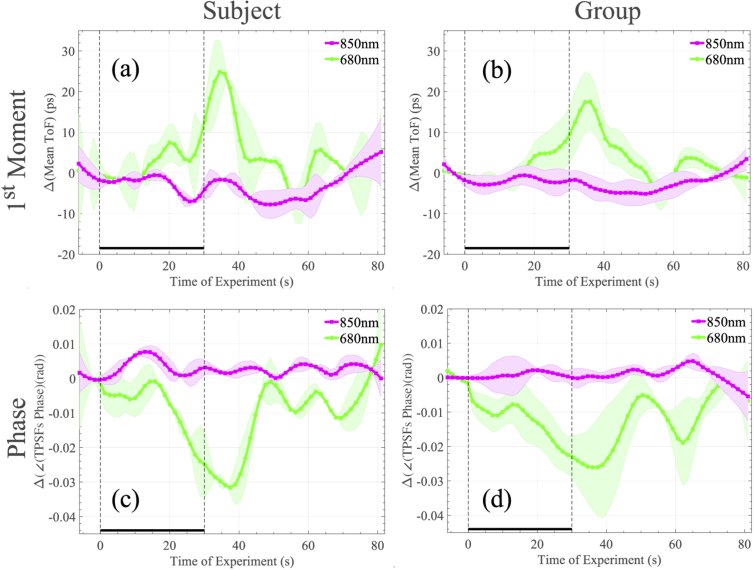
Collective TD results of the apnoea experiment. In (a) and (b) the mean ToF changes of an individual subject over 10 repetitions and the group’s average is shown, respectively. In (c) and (d) the phase angle changes of the FFT on the captured TPSFs of an individual subject over 10 repetitions and the group’s average is shown, respectively. The frequency bin at 236.22 MHz is illustrated. All changes have been plotted with respect to the baseline, defined as the beginning of the experiment. The two dashed vertical lines represent the beginning and end of the activation period.

As a further example of the rich data types which can be derived from the system, we show in Fig. 5 the changes in mean ToF and phase angle for each wavelength over the same experiment. Figures 5(a) and (b) show that the mean ToF at 680nm increases by circa 20ps at the peak of the ${\mathrm{HbO}}_{2}$ response. Figures 5(c) and (d) show that the phase angle of the light at 680nm and a modulation frequency of circa 236MHz is retarded by circa −0.03 rads over the same interval. In both cases, the 850nm source demonstrates very small changes. Direct interpretation of data in this format is challenging, a topic to which we shall return in our discussion.

Collectively, Figs. 3, 4, and 5 demonstrate that our system was capable of monitoring haemodynamic and TD related responses from the brain for all three subjects, which comply with the expected brain activity during this type of experiment.

### 3.3 SDMT experiment

This experiment was carried out closely following methodology described in [24,25]. In this type of experiment, the subject is asked to hand write symbols into blank squares paired with numbers as quickly as possible, by referring to a digit symbol key, outlined at the top of the sheet [25]. Because the task seeks correct, as well as fast responses, memorisation of the symbol-number association would inevitably enhance the subjects performance. Thus, this task is able to assess psychomotor, high-speed visuomotor/visuospatial tracking as well as attention and working memory.

In our case we used a custom graphical user interface (GUI) to implement the protocol, instead of the traditional paper-based approach. Subjects were presented with 9 symbols that corresponded to 9 digits in two rows at the top of the GUI and were asked to select the matching number from a larger set of symbols in the middle of the screen using a computer mouse. By implementing a computer-based method to perform SDMT, we focused more on the attention and working memory assessment, rather than on the visuomotor/visuospatial tracking. Using again a source detector separation of 30 mm, centred over F3 of the 10/20 EEG system (i.e. over the left frontal cortex region for cognitive function testing), the same three healthy adult subjects were tested in order to obtain group variances. The testing protocol consists of: (i) a 10 seconds baseline; (ii) followed by a 90 seconds activation period; and (iii) a 50 seconds relaxation period. For each subject 5 repetitions were performed and averaged. During the baseline and relaxation periods of the protocol, the computer monitor was showing a black background. During the activation stage, the software was loading the GUI automatically, based on a real-time software timer. The software timer also ensured that during all repetitions, the protocol remained time-consistent.

**Fig. 6.**
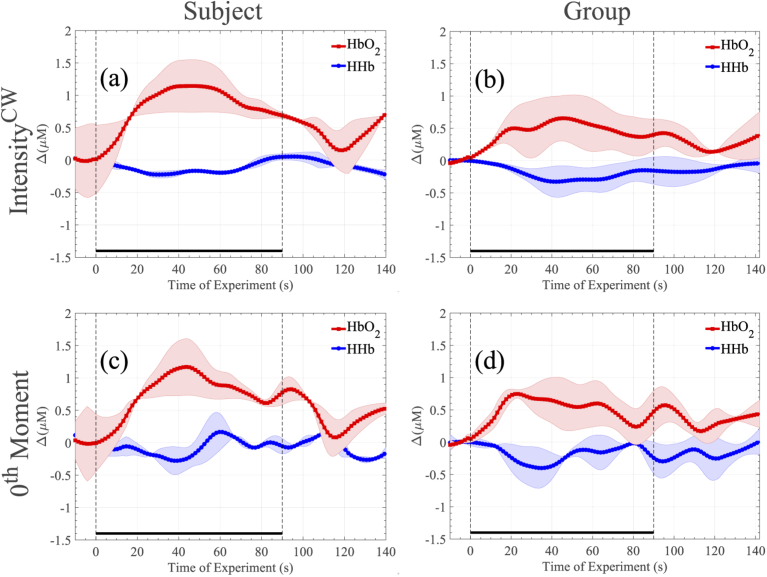
Haemodynamic responses of the SDMT experiment. In (a) and (c) the haemodynamic responses of an individual subject over 5 repetitions is shown while in (b) and (d) the group’s average is demonstrated. Figures (a) and (b) have been produced by calculating intensity as the total number of photons collected for each wavelength at each sample point. Figures (c) and (d) are based on the $0^{\text{th}}$-moment definition. All changes have been plotted with respect to the baseline, defined as the beginning of the experiment. The two dashed vertical lines represent the beginning and end of the activation period.

The haeomodynamic responses generated in this experiment are summarised in Fig. 6. Once again, Figs. 6(a) and (c) demonstrate the changes of an individual subject, computed based on Eq. (2) and Eq. (3), respectively. Figures 6(b) and (d) present the average changes of HHb and ${\mathrm{HbO}}_{2}$ of the group, computed based on Eq. (2) and Eq. (3), respectively. Both methods, in both cases, again generated very similar haemodynamic responses, as expected.

As Fig. 6 illustrates, after a stable baseline period, the activation period is giving rise to a distinct variation in HHb and ${\mathrm{HbO}}_{2}$ concentrations for all subjects, which tend to return to the initial baseline levels during the relaxation period. The need of the brain for more oxygen during the functional, cognitive activity is clearly indicated by the increase of ${\mathrm{HbO}}_{2}$ compared to a small decrease of HHb.

**Fig. 7.**
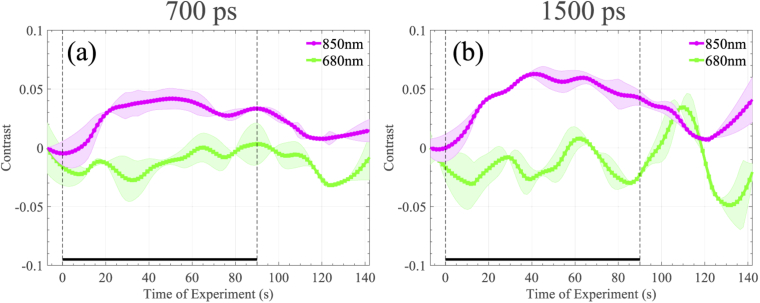
Contrast at 680 and 850nm for different time-gates, utilising a fixed width window equal to 300 ps. (a) demonstrates the contrast of an indicative subject during the SDMT experiment with 700 ps delay, while (b) shows contrast response of the same subject in the same experiment when the delay is 1500 ps. Average and standard deviation over 5 repetitions is shown in both cases.

We also consider two indicative contrast results using early time-gates before and after the mean ToF, as depicted in Fig. 7. Such measurements are particularly pertinent in this experiment, since SDMT is a cognitive task, and later arriving photons have greater sensitivity to the deeper regions (such as the brain) than those which arrive earlier (as described in [4,27]). Processing was performed as detailed in the apnoea experiment, where in Fig. 7(a) we present the contrast results for an early time gate of ${\text{t}}_{d}$ = 700 ps, and in Figure 7(b) we present the same measurement for a late time gate of ${\text{t}}_{d}$ = 1500 ps. Both figures demonstrate changes which correspond to the heamodynamic changes in Fig. 6, but it is clear that the contrast in the late gate is increased over that of the early gate, suggesting significant sensitivity to regions of functional activation.

## 4. Discussion

The experimental results presented in Section 3 provide an overview of the potential of the spread-spectrum technique to monitor in-vivo functional responses. In all cases, we were able to recover heamodynamic responses using intensity based measures. Direct interpretation of functional responses in time-domain data types such as mean-time, phase, and gated measurements is less well understood, but we have nonetheless demonstrated that these metrics can be extracted from the same data recorded by our instrument, supporting future investigations into the application of these measures in neuroscience.

Due to the nature of the excitation signal of our instrument, we expect the noise floor of our instrument to be higher compared to traditional pulsed-excitation systems. However, this inherent limitation does not seem to be an impediment for functional measurements. Indeed, it should be stressed that the selected experiments, and in particular the purely cognitive SDMT task, are considered challenging functional experiments, based on the small haemodynamic responses that they usually generate [25]. Figure 6 demonstrates changes of the HHb and ${\mathrm{HbO}}_{2}$ concentrations that vary between 0.2-0.5 µM and 0.5-1 µM, respectively between subjects. The presented cognitive task responses are almost one order of magnitude smaller compared to other common functional (motor) experiments such as hand-grip and finger-tapping [4,19,20]. Despite the challenging nature of the selected experiments, for each derived measurement the detected contrast is clearly above the noise level as measured by group-variance estimates. Indeed, the SDMT haemodynamic responses reported in [25] demonstrate a similar form to those we have recorded in this work, supporting the validity of our results. We further note that in the same experiment, the 50% contrast increase between the short and long time-dated data is in line with values reported in the literature [4], this is indicative of a deep functional response.

To date, we have focussed on the optimisation of the source side of our instrumentation in order to take full advantage of the spread-spectrum method. The 680 and 850 nm VCSELs of our setup are commercially available, with combined cost lower than $150. On the other hand, the cost of femto- or picosecond pulsed lasers can be prohibitive for non-clinical or research applications and, despite modern advancements in the field of laser fabrication, their size is also relatively large. Our system’s VCSELs have the size of a standard TO-46 package semiconductor, which is roughly 5.5×5.5×10 mm. Combined with the custom-made optical transceiver boards, the total size of each laser sources does not exceed dimensions of 50×15×10 mm. This could allow for the sources to be mounted directly on the subject to be imaged, removing the need for complex and slow optical routing and attenuation which inhibits rapid measurements in conventional pulsed systems. This characteristic lends itself to the development of high channel count imaging devices, as explored by other groups [30,31].

One obvious drawback of a VCSEL-SPAD based system compared to other contemporary pulsed-based TD NIRS system is multi-wavelength scanning. State-of-the-art pulsed-excitation systems utilise supercontinuum lasers that can allow multi-wavelength analysis of each sample [32]. With VCSELs, such approaches are not currently possible, given the single wavelength limitations of each device. However, the concept of VCSEL-SPAD systems is still in its infancy and future developments in VCSEL fabrication should permit the monolithic integration of high-speed multi-wavelength VCSELs for TD NIRS applications [33].

Encouraged by the potential of our novel instrument, future work will focus on the miniaturisation and integration of the detection side of the instrument. A particularly interesting avenue of research is to employ the high-performance FPGA, currently used to modulate and control the VCSEL-based sources of our system, to provide the functionality of the standalone TCSPC timing module. The high-speed processing capabilities of the Kintex 7 FPGA (similarly for other high-spec FPGAs) should permit operation as a high-resolution time-to-digital converter (TDC), with time-bin resolution below 100 ps. The current IRFs of our system demonstrate a FWHM of ∼600ps, which we believe to be a consequence of the performance of our SPCM modules at higher count rates. If desired, improved performance could be achieved by replacing these modules with the high time resolution variants such as the PDM series SPCM modules manufactured by Micro Photon Devices (Bolzano, Italy). Alternatively, further miniaturisation could be explored through the use of silicon photomultipliers in place of the SPAD modules.

Finally, the application of the spread spectrum technique permits a new method for multiplexing of multiple optical channels through the simultaneous use of different binary sequences, each decoded after transmission. This approach, code-division multiple access, is routinely employed in telecommunications applications.

## 5. Conclusion

We have presented the development of a dual-wavelength spread spectrum-based time-resolved system for fNIRS applications. For the first time, to our knowledge, we have measured functional responses from adult subjects using a spectroscopic, spread-spectrum system. The instrument was capable of detecting small haemodynamic changes from individual subjects, and provides repeatable estimates of time-domain information which, with appropriate analysis could provide measurements with enhanced depth sensitivity and rejection of superficial contamination.

The future developments we have outlined in this work provide a clear path to the development of highly miniaturised NIRS instruments, capable of providing quantitative measures of heamodynamic activity. The ultimate development of this technology could permit routine measurements of cerebral heamodynics with a device not considerably larger than the simple pulse-oxymeters employed routinely in clinical environments. Such a technology would allow new applications in clinical monitoring and neuroscience applications.
